# Temperature induces metabolic reprogramming in fish during bacterial infection

**DOI:** 10.3389/fimmu.2022.1010948

**Published:** 2022-09-15

**Authors:** Bin Sun, Boguang Sun, Beibei Zhang, Li Sun

**Affiliations:** ^1^ CAS Key Laboratory of Experimental Marine Biology, Institute of Oceanology, Center for Ocean Mega-Science, Chinese Academy of Sciences, Qingdao, China; ^2^ Institute of Ocean Research, Fujian Polytechnic Normal University, Fuqing, China; ^3^ Laboratory for Marine Biology and Biotechnology, Pilot National Laboratory for Marine Science and Technology (Qingdao), Qingdao, China; ^4^ College of Animal Science and Technology, Qingdao Agricultural University, Qingdao, China; ^5^ College of Earth and Planetary Sciences, University of Chinese Academy of Sciences, Beijing, China

**Keywords:** *Paralichthys olivaceus*, metabolism, edwardsiellosis, temperature, global warming, biomarker, *Edwardsiella*, bacterial infection

## Abstract

Water temperature elevation as a consequence of global warming results in increased incidence of bacterial disease, such as edwardsiellosis, in fish farming. Edwardsiellosis is caused by the bacterial pathogen *Edwardsiella tarda* and affects many farmed fish including flounder (*Paralichthys olivaceus*). Currently, the effect of temperature on the metabolic response of flounder to *E. tarda* infection is unclear. In this study, we found that compared to low temperature (15°C), high temperature (23°C) enhanced *E. tarda* dissemination in flounder tissues. To examine the impact of temperature on the metabolism of flounder induced by *E. tarda*, comparative metabolomics were performed, which identified a large number of metabolites responsive to *E. tarda* invasion and temperature alteration. During *E. tarda* infection, the metabolic profile induced by elevated temperature was mainly featured by extensively decreased amino acids and TCA intermediates such as succinate, a proven immune regulator. Further, 38 potential metabolite markers of temperature effect (MMTE) in association with bacterial infection were identified. When used as exogenous supplements, two of the MMTE, i.e., L-methionine and UDP-glucose, effectively upregulated the expression of pro-inflammatory cytokines and suppressed *E. tarda* infection in flounder leukocytes. Taken together, the results of this study indicate an important influence of temperature on the metabolism of flounder during bacterial infection, which eventually affects the survivability of the fish.

## Introduction

According to the Food and Agriculture Organization of the United Nations, the projected consequences of climate change, including rising temperature, increased risks of disease, altered precipitation pattern, ocean acidification and sea-level rise, impose immediate and long-term threats to the sustainability of aquaculture (1, 2). Fish and most other farmed aquatic animals are poikilothermic and therefore sensitive to the variation of ambient temperature (2). Rising water temperature *per se* and/or in combination with its potential environmental consequences, such as decreased level of dissolved oxygen, can exert multilayer impacts on various physiological processes of fish, notably metabolism and immune defense against pathogens (3, 4).

Elevated water temperatures have been associated with increased incidences of infectious disease in a number of farmed fish species, including flounder (*Paralichthys olivaceus*), channel catfish (*Ictalurus punctatus*) and Nile tilapia (*Oreochromis niloticus*) (5–8). Flounder (alternative names: Japanese flounder, olive flounder) is a species of marine teleost extensively cultured in Asia, in particular China, Japan, and Korea, and is extensively used as an experimental model in the studies on fish disease (9–13). The physiologically suitable temperatures of flounder range from 14°C to 23°C (14–16). In farmed flounder, edwardsiellosis is one of the most severe diseases, which occurs more frequently at high temperatures and can lead to mass mortality in numerous fish species farmed worldwide (17–20). The causative agent of edwardsiellosis is *Edwardsiella tarda*, a Gram-negative, facultative intracellular pathogenic bacterium (21–24). The pathogenicity of *E. tarda* depends on an armory of weapons including the type III secretion system (T3SS), type VI secretion system (T6SS), and other virulence factors (22, 23). Temperature is vital for the regulation of virulence in *E. tarda*. The bacterium senses the variation of environmental temperature *via* the two-component system PhoP-PhoQ (25). At 23°C-35°C, the sensor histidine kinase PhoQ phosphorylates the regulator PhoP, which subsequently activates the expression of T3SS, T6SS and other virulence genes, such as that encoding the anti-serum complement protease Sip1, to promote the onset of edwardsiellosis (25, 26).

Metabolism is considered a guiding force for immune cell activation and differentiation, and metabolites, such as succinate, can serve as signaling molecules in immune and inflammatory responses (27–30). In mammals, host metabolism is remodeled in response to bacterial infection (31). Some intracellular bacteria, such as *Mycobacterium tuberculosis*, *Legionella pneumophila*, *Brucella abortus*, *Chlamydia trachomatis*, and *C. pneumoniae*, are able to reprogram the metabolism of host cells, whereby eliciting a phenomenon termed Warburg-like effect (32). Warburg effect is known as a hallmark of cancer cells, in which glucose uptake and glycolysis are accelerated, and the final product of glycolysis, pyruvate, is converted to lactate in the cytosol, rather than being routed to oxidative phosphorylation in the mitochondria as seen in normal non-proliferating cells (33). Pathogenic bacteria-induced Warburg-like metabolism in host cells accumulates the intermediates of glycolysis and tricarboxylic acid (TCA) cycle, whereby enhancing the anabolism of amino acids, nucleotides and lipids, which support host immunity and also serve as nutrients for the pathogen (32, 34). Considering the essential role of metabolism in shaping immunity (28, 35), metabolites have been employed to regulate host immunity and combat bacterial infection (36). In teleost, the metabolomic approach has been used to identify the crucial metabolic biomarkers that benefit host survival during bacterial infection (37, 38). In crucian carp (*Carassius auratus*), tilapia (*Oreochromis niloticus*) and zebrafish (*Danio rerio*), exogenously supplemented metabolites could enhance the survival of fish during *E. tarda* infection (5, 39, 40).

Increasing evidences indicate that in flounder, *E. tarda* infection elicits profound immune responses through an elaborate network composed of immune molecules such as proteins and regulatory RNAs (41–43). However, the metabolic response of flounder to *E. tarda* infection, especially under different temperatures, remains unclear. In this study, we investigated the metabolomic profiles of flounder induced by *E. tarda* infection at high and low temperatures, and identified potential biomarkers for temperature-regulated metabolic reprogramming in response to bacterial infection. Our results revealed a metabolic connection between temperature elevation and edwardsiellosis in flounder, and indicated a risk of increased fish disease as a result of global warming.

## Materials and methods

### Fish and bacteria

Flounder (*Paralichthys olivaceus*) averaging 250 g in weight were purchased from a local fish farm in Qingdao, China. The fish were maintained at ~15°C in aerated seawater and fed daily with a commercial feed. Prior to experiments, the fish were acclimatized for a week and verified to be clinically healthy as reported previously (44). For tissue collection, flounder were euthanized with tricaine methane sulfonate (Sigma, St. Louis, MO, USA) as described previously (45). *Edwardsiella tarda* TX1 was isolated from diseased founder (46).

### Experimental grouping, infection and sampling

Flounder were randomly divided into four groups (9 fish/group), namely, the low temperature control group (LC), the high temperature control group (HC), the low temperature infection group (LI), and the high temperature infection group (HI). All fish were acclimatized at 15°C for one week. Then the LC and LI groups were maintained continuously at 15°C, while the HC and HI groups were maintained at the temperature that increased by 1°C per day until 23°C. The HC and HI groups were maintained at 23°C for one week. Meanwhile, *E. tarda* was grown in Leibovitz L-15 medium (Gibico, Grand Island, NY, USA) at 15°C or 23°C till OD_600_ ≈ 0.4. The bacteria were collected by centrifugation, washed with PBS and suspended in PBS to a final concentration of 1 x 10^8^ CFU/mL. The LI and HI groups were intramuscularly injected with 100 μL bacterial suspension of the 15°C and 23°C culture, respectively; the LC and HC groups were injected with a same volume of PBS. After injection, the LC and LI groups were maintained at 15°C for 24 h; the HC and HI groups were maintained at 23°C for 24 h. Spleen was aseptically collected from the fish of each group. One portion of the spleen sample was used to analyze bacterial recovery by plate count as reported previously (47). The rest of the sample was frozen in liquid nitrogen and used for metabolomic analysis.

### Liquid chromatography/tandem mass spectrometry (LC-MS/MS)

A total of 36 samples (9 samples/group) were subjected to metabolomic LC-MS/MS analysis with an UHPLC-Q-TOF-MS approach. For each sample, 60 mg tissue was homogenized in 200 μL water. Then the metabolites were extracted with 800 μL methanol: acetonitrile solution (1:1, v/v) and lyophilized using a vacuum centrifuge at 4°C. The extract was dissolved in 100 μL acetonitrile/water (1:1, v/v), and 2 μL of each sample was added onto an ACQUITY UPLC BEH Amide column (1.7 μm, 2.1 mm× 100 mm) (Waters, Wexford, Ireland) for hydrophilic interaction liquid chromatography (HILIC) with Agilent 1290 Infinity LC ultrahigh performance liquid chromatography (UHPLC) system. The automatic sampler was maintained at 4°C during the LC process. The separation was performed at 25°C, 0.3 mL/min, with the gradient of solvent A (25 mM ammonium acetate and 25 mM ammonium hydroxide in water) and solvent B (acetonitrile) as follows: 0-1 min, 95% B; 1-14 min, 95%-65% B; 14-16 min, 65%-40% B; 16-18 min, 40% B; 18-18.1 min, 40%-95%; 18.1-23 min, 95% B. A tandem MS/MS was coupled to the LC separation using a Triple TOF 5600 mass spectrometer (AB SCIEX). The electrospray ionization (ESI) source conditions were set as follows: Ionization modes: negative and positive; Ion Source Gas1 (Gas1), 60; Ion Source Gas2 (Gas2), 60; curtain gas (CUR), 30; source temperature, 600°C; Ion Spray Voltage Floating (ISVF), ± 5500 V. The parameters of TOF MS were set as follows: m/z range, 60-1000; accumulation time, 0.20 s/spectra. The secondary MS was performed with the information dependent acquisition (IDA) approach in the high sensitivity mode. The parameters were set as follows: m/z range, 25-1000; accumulation time, 0.05 s/spectra; collision energy (CE), 35 V ± 15 eV; declustering potential (DP): ± 60 V.

### Metabolomic data processing and analysis

The raw data of LC-MS/MS was converted to the MzXML format using ProteoWizard (https://proteowizard.sourceforge.io) and imported to the XCMS (https://xcmsonline.scripps.edu) software for retention time correction, peak detection and matching. The metabolites were identified based on exact mass (< 25 ppm) and compound spectrum matching with a self-built database constructed by Shanghai Applied Protein Technology Co., Ltd. After signal normalization, the data were processed by SIMCA-P 14.1 (Umetrics, Umea, Sweden) for Unsupervised Principal Component Analysis (PCA), Supervised Partial Least Squares Discriminant Analysis (PLS-DA) and Orthogonal Partial Least Squares Discriminant Analysis (OPLS-DA). The variable Importance for the Projection (VIP) value derived from OPLS-DA was used to evaluate the contribution of a metabolite to its discriminative grouping for screening the potential metabolic biomarkers upon different treatments (thermal stress and/or bacterial infection). The statistical significance was determined with an unpaired Student’s *t* test. VIP value >1, *p*<0.05 and |fold change| > 2 were set as the screening criteria to identify significantly differential metabolites (SDMs). The metabolite category, Venn diagram and heat map analysis of the SDMs were performed using the platform of Shanghai Applied Protein Technology Co., Ltd (http://cloud.aptbiotech.com/#/main-page). Pathway enrichment analysis was conducted using MetaboAnalyst 5.0 (https://www.metaboanalyst.ca/) and the KEGG database (https://www.kegg.jp) as reported previously (5, 48). Pathways with *p* value < 0.05 were considered significantly enriched.

### Isolation of the flounder spleen leukocytes

Flounder spleen leukocytes were isolated as described previously with minor modification (49). In brief, founder spleen was collected aseptically and placed onto a cell strainer (BD Falcon, Franklin Lakes, NJ, USA) on top of a 50-mL tube. Then the spleen was cut to pieces and gently grinded in L-15 medium with a syringe plunger, during which process, the cells were separated by passing through the strainer. The cell suspension was loaded onto a Percoll (GE Healthcare, Uppsala, Sweden) solution with the density of 1.070 g/mL and centrifuged at 400 × g for 10 min. The leukocyte layer was collected with a syringe and suspended in L-15 medium.

### Cellular infection assay

To assess the effects of the selected metabolites on *E. tarda* infection to flounder leukocytes, the metabolites (dissolved in L-15 medium) were added to the cells in 1.5-mL Eppendorf tubes to a final concentration of 0.5 mM (50). The control group was added with L-15 medium. The cells (1 x 10^7^ cells/mL, 1 mL/tube, 3 replicates/group) were incubated at 23°C for 6 h. Meanwhile, *E. tarda* was grown in Luria–Bertani (LB) medium at 28°C to an OD_600_ of 0.7 and suspended in L-15 medium to a final concentration of 1×10^9^ CFU/ml. The above flounder leukocytes were infected with *E. tarda* at an MOI of 5:1 and incubated at 23°C for 2 h with constant slow rotation. Then the leukocytes were centrifuged at 400 × g for 5 min and gently washed with PBS for 3 times. The leukocytes were divided into two portions: one portion was lysed with 1% Triton X-100 and subjected to bacterial recovery analysis by plate count. The other portion was used for the quantitative real time reverse transcription PCR (qRT-PCR) assay. All experiments were performed in triplicate.

### qRT-PCR assay

RNA was extracted using the RNA-easy Isolation Reagent (Vazyme, Nanjing, China). cDNA was obtained by reverse transcription using the ReverTra Ace qPCR RT Master Mix with gDNA remover (Toyobo, Osaka, Japan). qRT-PCR was performed in technical duplicate (three biological replicates for a treatment group) using the ChamQ Universal SYBR qPCR Master Mix (Vazyme, Nanjing, China) in a QuantStudio 3 Real-Time PCR System (Thermo Fisher Scientific, CA, USA). Gene expression was analyzed with the comparative threshold cycle (2^−ΔΔC^ *^t^*) method using *β-actin* as the internal control (51). The primers are listed in **Table 1**.

**Table 1:** Primers used for qRT-PCR.

Gene name	Forward primer(5′-3′)	Reverse primer(5′-3′)
TNF-α	CTGGTGTGGAAGAACGACGA	CGTGAGGTGTTTTTCCGCTG
IL-1β	GTCCACCTATGTGCACCCTT	CATTTGTTCTCGACACGCTCC
IL-6	CTCCAGTCGAATACGAGCCC	ACTCTTTCTGGTGGTGAGCG
IL-8	GCCTGAGAAGCCTAGGAGTG	TGACTCTCTTCACCCACGGA
IL-27β	TGGCTGCGATGTTGGTTACT	TTCAGGCCAGGAGCAAAGAG
β-actin	GCACGGTATTGTGACCAACTGG	CAGGGGAGCCTCTGTGAGC

### Statistical analysis

For the cellular infection and qRT-PCR, data were analyzed with Student’s *t* test, and statistical significance was defined as *p* < 0.05.

## Results

### Temperature has a significant impact on *E. tarda* infection

To examine the effect of temperature on *E. tarda* infection in flounder, the infection study was performed with flounder acclimatized at 15°C (low temperature) or 23°C (high temperature) (**Figure S1A**). The fish acclimatized at each temperature were divided into the infected and control groups. The infected groups were named LI (low temperature infection) or HI (high temperature infection), and the respective control groups were named LC (low temperature control) or HC (high temperature control). At 24 hpi, the spleen bacterial load in the HI group was 51.3-fold of that in the LI group (**Figure S1B**), indicating a strong effect of temperature on the ability of flounder to block bacterial infection.

### Metabolite profiles of flounder infected by *E. tarda* at different temperatures

**Figure 1:**
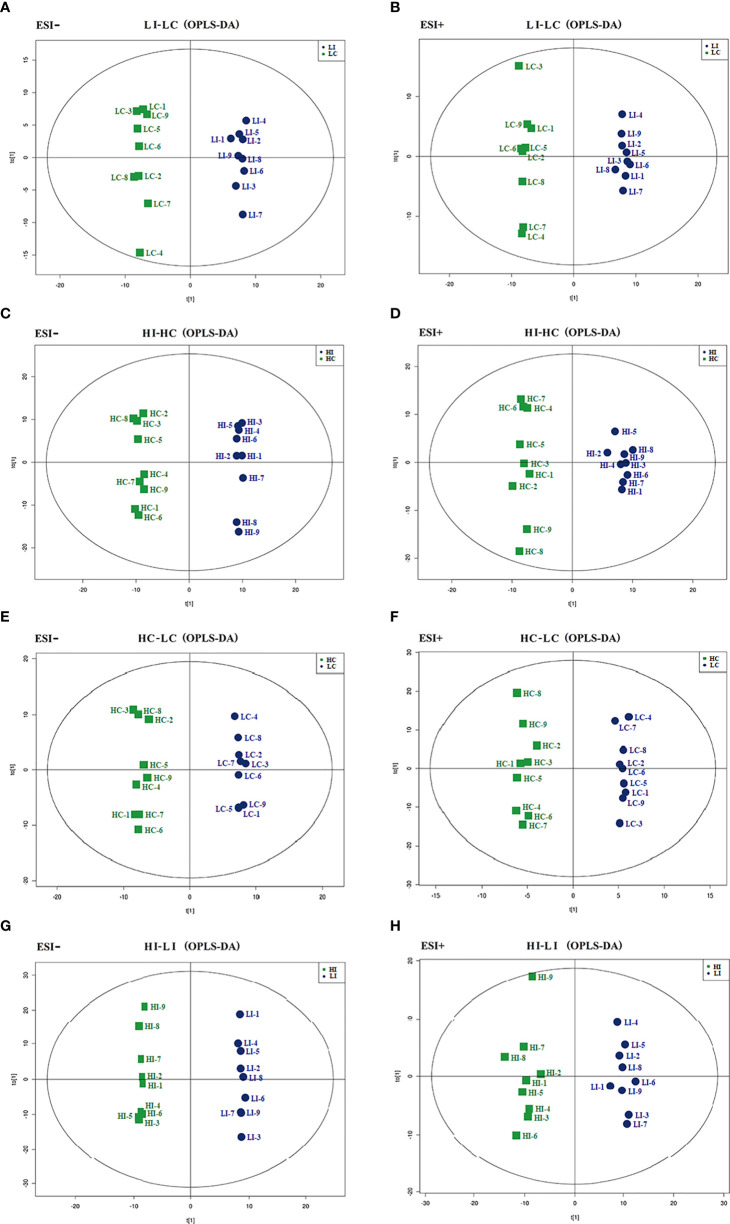
OPLS-DA score plots. The OPLS-DA score plots of LI−LC **(A, B)**, HI−HC **(C, D)**, HC−LC **(E, F)** and HI−LI **(G, H)** were obtained in negative **(A, C, E, G)** and positive ionization modes **(B, D, F, H)**.

Comparative metabolomic analysis was conducted to examine the metabolite response to infection and temperature. A total of 9733 and 7428 MS peaks were identified in the negative and positive ionization modes, respectively. Four comparisons, i.e., LI versus LC (LI−LC), HI versus HC (HI−HC), HC versus LC (HC−LC), and HI versus LI (HI−LI) were conducted to examine the metabolic changes in response to *E. tarda* or temperature. Specifically, LI−LC and HI−HC assessed the change caused by bacterial infection at 15°C and 23°C, respectively; HC−LC assessed the change caused by temperature in the absence of infection; HI−LI assessed the change caused by temperature under the condition of *E. tarda* infection. OPLS-DA discrimination showed clear metabolic differentiation in both the negative and positive ionization modes between LI and LC, HI and HC, HC and LC, and HI and LI (**Figure 1**), thus establishing a robust model for metabolite classification. Based on the OPLS-DA, VIP > 1 and *p* < 0.05 were set as the screening criteria for differential metabolites, and a total of 89, 74, 23, and 101 differential metabolites were identified in LI−LC, HI−HC, HC−LC, and HI−LI, respectively (**Table S1**-**S4**).

### *E. tarda*-responsive metabolites

**Figure 2:**
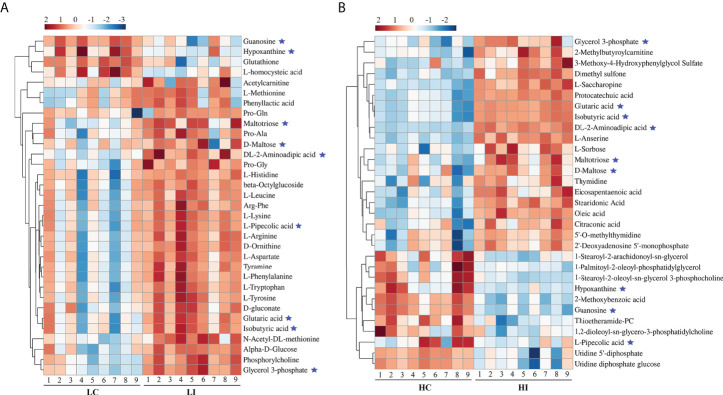
Heatmap representation of the SDMs in LI−LC and HI−HC. The relative metabolite abundance in LC−LI **(A)** and HC−HI **(B)** are shown. Red and blue represent up- and down-regulation, respectively. ★, metabolites occurring in both LI−LC and HI−HC.

For the identification of Significantly Differential Metabolites (SDMs), VIP > 1, *p* < 0.05 and |fold change| > 2 were set as the threshold. Thirty-three and 31 SDMs were identified in LI−LC and HI−HC, respectively (**Table S1**, **S2**). Nine SDMs, i.e., guanosine, hypoxanthine, maltotriose, D-maltose, DL-2-aminoadipic acid, L-pipecolic acid, glutaric acid, isobutyric acid, and glycerol 3-phosphate, were shared by LI−LC and HI−HC (**Figure 2**, **Figure S2**). The 33 SDMs in LI−LC were categorized into the top 10 KEGG pathways of Aminoacyl-tRNA biosynthesis, Phenylalanine, tyrosine and tryptophan biosynthesis, Phenylalanine metabolism, Lysine degradation, Arginine biosynthesis, Histidine metabolism, beta-Alanine metabolism, D-Arginine and D-ornithine metabolism, Valine, leucine and isoleucine biosynthesis, and Ubiquinone and other terpenoid-quinone biosynthesis (**Figure S3A**, **Table S5**). The 31 SDMs in HI-HC were categorized into the top 10 KEGG pathways of Lysine degradation, Starch and sucrose metabolism, Purine metabolism, Biosynthesis of unsaturated fatty acids, Ascorbate and aldarate metabolism, Pyrimidine metabolism, alpha-Linolenic acid metabolism, Histidine metabolism, Glycerolipid metabolism, and Pentose and glucuronate interconversions (**Figure S3B**, **Table S6**).

### Temperature-responsive metabolites

**Figure 3:**
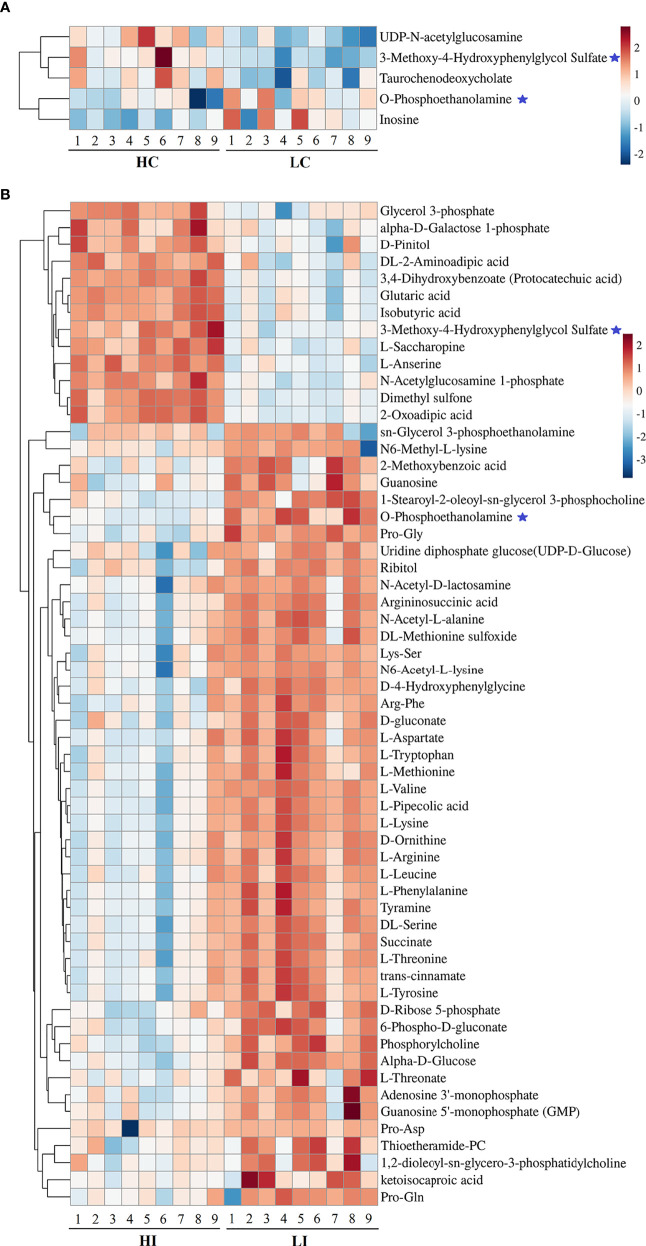
Heatmap representation of the SDMs in HC−LC and HI−LI. The relative metabolite abundance in HC−LC **(A)** and HI−LI **(B)** are shown. Red and blue represent up- and down-regulation, respectively. ★, metabolites occurring in both HC−LC and HI−LI.

**Figure 4:**
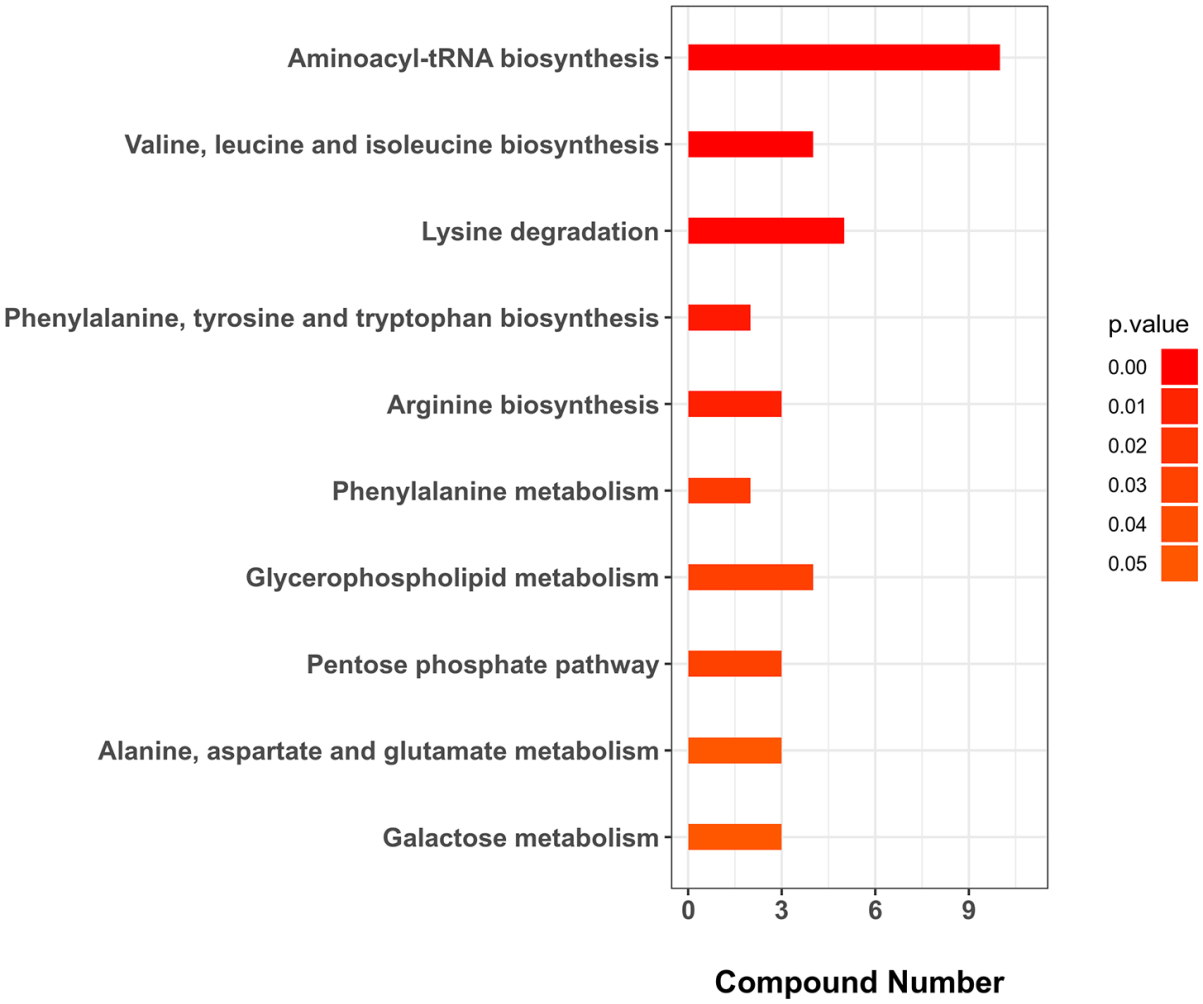
KEGG enrichment (top 10) of the unique SDMs in HI−LI. The *p*-value was set as < 0.05.

Five and 59 SDMs were identified in HC−LC and HI−LI, respectively (**Table S3**, **S4**, **Figure S4** **)**. As shown in **Figure 3** and **Figure S5**, two SDMs, i.e., 3-methoxy-4-hydroxyphenylglycol sulfate and O-phosphoethanolamine, were shared by HC−LC and HI−LI. Three SDMs, i.e., UDP-N-acetylglucosamine, taurochenodeoxycholate, and inosine, were exclusively identified in HC−LC. Fifty-seven SDMs were exclusively identified in HI−LI (**Table 2**), of which, 47.37% were amino acids, 14.04% were other organic acids, 14.04% were carbohydrates, 8.77% were lipids, and 5.26% were nucleotides (**Figure S6A**). Thirteen and 44 of these metabolites were increased and decreased in abundance, respectively (**Figure S6B**). The 57 SDMs were categorized into the metabolic pathways of Aminoacyl-tRNA biosynthesis, Valine, leucine and isoleucine biosynthesis, Lysine degradation, Phenylalanine, tyrosine and tryptophan biosynthesis, Arginine biosynthesis, Phenylalanine metabolism, Glycerophospholipid metabolism, Pentose phosphate pathway, Galactose metabolism, and Alanine, aspartate and glutamate metabolism (**Figure 4**, **Table 3**).

**Table 2:** List of the 57 SDMs in HI−LI. "+" and "-" indicate up- and down-regulations, respectively, at high temperature compared to low temperature.

Metabolites	VIP	Fold change	*p*-value	m/z	rt(s)
L-Anserine	3.68	+5.69	1.61E-06	241.13	819.70
Dimethyl sulfone	3.13	+5.12	5.81E-07	226.98	725.40
alpha-D-Galactose 1-phosphate	1.08	+4.27	1.25E-02	241.01	720.80
Protocatechuic acid	1.89	+3.41	8.34E-08	153.02	689.08
D-Pinitol	1.06	+3.16	2.07E-03	261.03	944.86
DL-2-Aminoadipic acid	1.37	+3.12	6.83E-05	144.06	798.12
L-Saccharopine	1.12	+2.77	3.02E-05	275.12	819.47
Glycerol 3-phosphate	1.37	+2.72	1.35E-04	171.01	777.84
Glutaric acid	8.04	+2.39	1.23E-07	131.03	689.12
Isobutyric acid	2.47	+2.29	1.62E-06	87.04	689.13
N-Acetylglucosamine 1-phosphate	1.15	+2.29	2.72E-06	300.05	797.92
2-Oxoadipic acid	11.49	+2.14	5.06E-08	141.02	666.69
D-Ribose 5-phosphate	2.51	-2.02	3.03E-04	289.03	836.59
sn-Glycerol 3-phosphoethanolamine	1.18	-2.03	4.04E-02	216.06	778.73
N-Acetyl-L-alanine	2.01	-2.04	1.27E-04	130.05	446.69
DL-Serine	3.94	-2.05	1.05E-04	104.03	678.82
Pro-Asp	3.91	-2.07	2.09E-04	291.12	914.23
Thioetheramide-PC	5.74	-2.16	1.90E-02	758.58	142.21
ketoisocaproic acid	2.82	-2.16	1.85E-02	129.05	222.04
Phosphorylcholine	1.42	-2.17	7.35E-05	242.08	711.53
L-Aspartate	6.01	-2.18	2.77E-03	132.03	725.44
Lys-Ser	1.72	-2.24	1.54E-04	275.17	907.40
SOPC	1.36	-2.29	1.82E-05	787.61	308.38
Succinate	3.02	-2.30	9.14E-05	136.06	598.66
L-Threonine	5.11	-2.30	2.44E-04	118.05	635.86
2-Methoxybenzoic acid	2.18	-2.35	2.97E-03	152.05	522.31
D-Ornithine	2.03	-2.38	7.78E-04	131.08	968.69
L-Valine	5.66	-2.40	2.05E-06	118.08	597.20
UDP-Glucose	1.22	-2.40	3.71E-03	565.05	801.17
Pro-Gln	2.47	-2.43	1.82E-03	304.15	886.65
Argininosuccinic acid	2.36	-2.44	1.50E-05	289.11	854.80
trans-cinnamate	2.32	-2.44	5.54E-04	147.04	456.64
6-Phospho-D-gluconate	1.18	-2.44	6.99E-04	275.02	886.42
Guanosine	1.84	-2.46	1.24E-02	282.08	480.56
N-Acetyl-D-lactosamine	1.33	-2.47	1.64E-03	404.12	661.05
L-Leucine	16.75	-2.47	5.16E-04	130.09	466.98
N6-Methyl-L-lysine	1.58	-2.48	8.31E-04	161.13	1070.15
N6-Acetyl-L-lysine	3.62	-2.49	5.18E-06	187.11	636.67
L-Tyrosine	7.30	-2.53	5.91E-04	180.07	538.85
L-Arginine	4.47	-2.55	1.73E-03	173.10	966.58
L-Phenylalanine	9.55	-2.66	1.44E-03	166.08	514.27
Tyramine	7.51	-2.67	9.72E-04	120.08	514.45
Alpha-D-Glucose	1.40	-2.71	5.03E-06	179.06	467.09
Adenosine 3’-monophosphate	1.87	-2.77	1.53E-02	346.05	731.88
D-4-Hydroxyphenylglycine	1.30	-2.85	1.13E-04	168.07	753.34
L-Tryptophan	3.53	-2.89	4.20E-03	203.08	458.97
L-Threonate	2.33	-2.90	1.59E-02	135.03	554.94
DL-Methionine sulfoxide	4.41	-2.93	4.19E-04	164.04	662.89
L-Methionine	4.28	-3.06	3.67E-03	148.04	506.72
Guanosine 5’-monophosphate (GMP)	1.32	-3.09	4.44E-02	362.05	794.25
1,2-dioleoyl-sn-glycero-3-phosphatidylcholine	3.13	-3.10	4.20E-02	786.61	138.88
Arg-Phe	1.40	-3.17	3.80E-04	286.17	602.04
L-Pipecolic acid	6.35	-3.19	3.11E-05	130.08	1042.18
Ribitol	1.04	-3.57	1.33E-05	151.06	409.91
D-gluconate	4.47	-3.66	9.93E-04	195.05	665.90
L-Lysine	7.40	-4.47	6.44E-05	145.10	984.57
Pro-Gly	1.16	-5.47	1.08E-04	345.17	698.36

**Table 3:** Top 10 KEGG enriched pathways of the unique SDMs in HI−LI. The *p*-value was set as < 0.05. Up and down arrows indicate up- and down-regulations, respectively.

Metabolic pathway	Hits	*p*-value	Metabolites
Aminoacyl-tRNA biosynthesis	10	8.77E-07	L-Valine (↓), L-Phenylalanine (↓), L-Arginine (↓), L-Aspartate (↓), L-Methionine (↓), L-Lysine (↓), L-Leucine (↓), L-Tryptophan (↓), L-Tyrosine (↓), L-Threonine (↓)
Valine, leucine and isoleucine biosynthesis	4	5.37E-05	L-Threonine (↓), L-Leucine (↓), L-Valine (↓), Ketoisocaproic acid (↓)
Lysine degradation	5	8.00E-04	L-Lysine (↓), L-Saccharopine (↓), DL-2-Aminoadipic acid (↑), 2-Oxoadipic acid (↑), L-Pipecolic acid (↓)
Phenylalanine, tyrosine and tryptophan biosynthesis	2	5.53E-03	L-Phenylalanine (↓), L-Tyrosine (↓)
Arginine biosynthesis	3	8.21E-03	L-Arginine (↓), L-Aspartate (↓), Argininosuccinic acid (↓)
Phenylalanine metabolism	2	2.38E-02	L-Phenylalanine (↓), L-Tyrosine (↓)
Glycerophospholipid metabolism	4	2.85E-02	Phosphorylcholine (↓), Choline phosphate (↓), Glycerol 3-phosphate (↑), sn-Glycerol 3-phosphoethanolamine (↓)
Pentose phosphate pathway	3	2.91E-02	D-Ribose 5-phosphate (↓), 6-Phospho-D-gluconate (↓), D-gluconate (↓)
Galactose metabolism	3	4.97E-02	Alpha-D-Glucose (↓), UDP-glucose (↓), alpha-D-Galactose 1-phosphate (↑)
Alanine, aspartate and glutamate metabolism	3	4.97E-02	L-Aspartate (↓), Argininosuccinic acid (↓), Succinate (↓)

### Potential Metabolite Markers of Temperature Effect (MMTE) during bacterial infection

**Figure 5:**
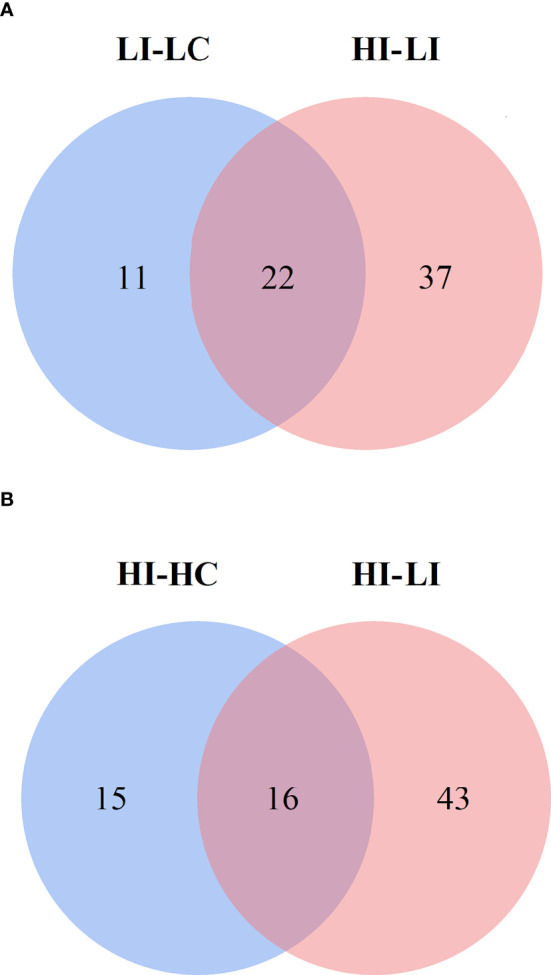
Venn diagrams showing the intersection between the SDMs in LI−LC and HI−LI **(A)** and the SDMs in HI−HC and HI−LI **(B)**. The blue circles represent LI−LC **(A)** and HI−HC **(B)**. The pink circles represent HI−LI.

To identify the SDMs that could potentially serve as the biomarkers indicative of the effect of temperature on metabolism during *E. tarda* infection, the SDMs of LI−LC versus HI−LI and the SDMs of HI−HC versus HI−LI were compared. Twenty-two common SDMs were identified between LI−LC and HI−LI (**Figure 5A**) and named Group-1 MMTE. These metabolites exhibited fold changes ranging from -2.24 to +5.70 (**Table 4**). They were categorized into the metabolic pathways of Aminoacyl-tRNA biosynthesis, Phenylalanine, tyrosine and tryptophan biosynthesis, Lysine degradation, Phenylalanine metabolism, Arginine biosynthesis, D-Arginine and D-ornithine metabolism, Glycerophospholipid metabolism, Valine, leucine and isoleucine biosynthesis, Tyrosine metabolism, and Ubiquinone and other terpenoid-quinone biosynthesis (**Figure S7**, **Table S7**). Sixteen common SDMs were identified between HI−HC and HI−LI (**Figure 5B**) and named Group-2 MMTE. These metabolites exhibited fold changes ranging from -7.87 to +26.03 (**Table 5**). They were categorized into the metabolic pathways of Lysine degradation, Linoleic acid metabolism, Glycerophospholipid metabolism, Ascorbate and aldarate metabolism, alpha-Linolenic acid metabolism, Histidine metabolism, Glycerolipid metabolism, Starch and sucrose metabolism, Pentose and glucuronate interconversions, and Galactose metabolism (**Figure S8**, **Table S8**).

**Table 4:** List of the 22 SDMs shared between LI−LC and HI−LI. "+" and "-" indicate up- and down-regulations, respectively.

Metabolites	LI−LC			HI−LI		
	VIP	Fold change	*p*-value	VIP	Fold change	*p*-value
DL-2-Aminoadipic acid	1.19	+5.70	4.98E-03	1.37	+3.12	6.83E-05
L-Methionine	4.62	+4.02	1.78E-02	4.28	-3.06	3.67E-03
L-Tryptophan	3.91	+3.07	3.54E-03	3.53	-2.89	4.20E-03
L-Lysine	7.27	+2.93	8.73E-04	7.40	-4.47	6.44E-05
Alpha-D-Glucose	1.51	+2.72	3.22E-04	1.40	-2.71	5.03E-06
L-Arginine	4.91	+2.64	1.06E-03	4.47	-2.55	1.73E-03
D-Ornithine	2.29	+2.59	2.62E-04	2.03	-2.38	7.78E-04
Pro-Gly	1.16	+2.58	2.46E-03	1.16	-5.47	1.08E-04
Phosphorylcholine	1.65	+2.55	5.94E-05	1.42	-2.17	7.35E-05
L-Aspartate	6.90	+2.50	1.41E-03	6.01	-2.18	2.77E-03
Glycerol 3-phosphate	1.05	+2.48	1.19E-05	1.37	+2.72	1.35E-04
D-gluconate	4.17	+2.45	1.08E-02	4.47	-3.66	9.93E-04
Tyramine	8.77	+2.37	2.35E-03	7.51	-2.67	9.72E-04
L-Tyrosine	7.65	+2.37	1.01E-03	7.30	-2.53	5.91E-04
L-Phenylalanine	11.28	+2.35	3.46E-03	9.55	-2.66	1.44E-03
L-Pipecolic acid	6.74	+2.29	7.53E-04	6.35	-3.19	3.11E-05
Pro-Gln	2.59	+2.08	7.83E-03	2.47	-2.43	1.82E-03
Arg-Phe	1.44	+2.06	4.10E-03	1.40	-3.17	3.80E-04
Glutaric acid	4.37	+2.05	1.53E-03	8.04	+2.39	1.23E-07
Isobutyric acid	1.39	+2.02	2.64E-03	2.47	+2.29	1.62E-06
L-Leucine	16.12	+2.01	3.46E-03	16.75	-2.47	5.16E-04
Guanosine	3.57	-2.24	2.98E-04	1.84	-2.46	1.24E-02

**Table 5:** List of the 16 SDMs shared between HI−HC and HI−LI. "+" and "-" indicate upregulation and downregulation, respectively.

Metabolites	HI−HC			HI−LI		
	VIP	Fold change	*p*-value	VIP	Fold change	*p*-value
DL-2-Aminoadipic acid	1.83	+26.03	4.89E-08	1.37	+3.12	6.83E-05
L-Anserine	4.30	+8.54	4.75E-07	3.68	+5.69	1.61E-06
Protocatechuic acid	1.95	+7.22	3.54E-09	1.89	+3.41	8.34E-08
Glutaric acid	8.99	+6.42	6.96E-11	8.04	+2.39	1.23E-07
Isobutyric acid	2.80	+6.05	8.19E-10	2.47	+2.29	1.62E-06
L-Saccharopine	1.17	+4.16	1.70E-06	1.12	+2.77	3.02E-05
Dimethyl sulfone	3.24	+3.89	2.02E-06	3.13	+5.12	5.81E-07
3-Methoxy-4-Hydroxyphenylglycol Sulfate	3.81	+2.29	9.79E-03	4.61	+3.56	6.21E-04
Glycerol 3-phosphate	1.18	+2.10	7.95E-04	1.37	+2.72	1.35E-04
Thioetheramide-PC	10.43	-3.15	3.13E-03	5.74	-2.16	1.90E-02
L-Pipecolic acid	1.08	-3.30	4.35E-02	6.35	-3.19	3.11E-05
1,2-dioleoyl-sn-glycero-3-phosphatidylcholine	3.86	-3.35	3.84E-02	3.13	-3.10	4.20E-02
SOPC	2.22	-3.52	3.30E-03	1.36	-2.29	1.82E-05
2-Methoxybenzoic acid	4.30	-3.81	1.19E-05	2.18	-2.35	2.97E-03
UDP-Glucose	2.14	-5.15	1.94E-06	1.22	-2.40	3.71E-03
Guanosine	1.20	-7.87	3.18E-05	1.84	-2.46	1.24E-02

### The effects of selected MMTE on *E. tarda* infection and flounder immune response

**Figure 6:**
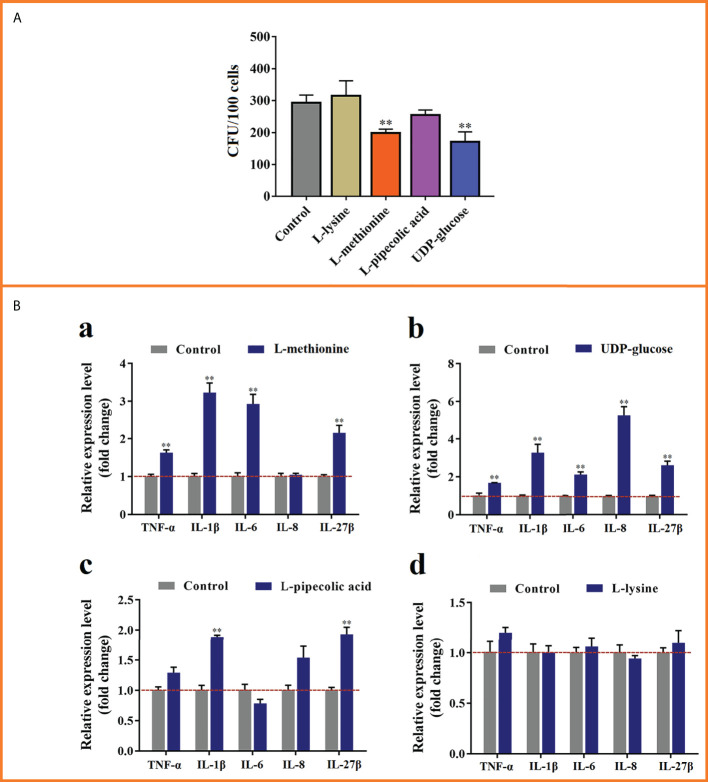
The effects of selected metabolites on *Edwardsiella tarda* infection and cytokine expression. **(A)** Flounder leukocytes were pre-incubated with L-lysine, L-methionine, L-pipecolic acid, or UDP-glucose for 6 h, and then infected with *E tarda* for 2h Cell-infected bacteria (shown as Colony Forming Unit, CFU) were determined by plate count. **(B)** The cells treated above were subjected to qRT-PCR to analyze the expression of TNF-α, IL-1β, IL-6, IL-8, and IL-27β. **, *p* < 0.01. For both panels, values are the means ± SEM of three independent experiments. **, *p* < 0.01.

To examine the effects of MMTE on *E. tarda* infection, four metabolites, i.e., L-lysine and L-methionine from Group-1 MMTE, UDP-glucose from Group-2 MMTE, and L-pipecolic acid shared by Group-1 and Group-2 MMTE, were selected and tested for their effects on *E. tarda* infection of flounder leukocytes. The results showed that L-methionine and UDP-glucose significantly suppressed the infection of *E. tarda* into flounder cells, while L-lysine and L-pipecolic acid had no obvious effect (**Figure 6A**). qRT-PCR analysis of the pro-inflammatory gene expression in these cells showed that L-methionine significantly enhanced the expression of IL-1β (3.23-fold), IL-6 (2.93-fold), IL-27β (2.15-fold), and TNF-α (1.63-fold) (**Figure 6** **Ba**); UDP-glucose significantly increased the expression of IL-1β (3.27-fold), IL-6 (2.14-fold), IL-8 (5.27-fold) and IL-27β (2.63-fold) (**Figure 6** **Bb**). L-pipecolic acid also significantly enhanced the expression of IL-1β (1.89-fold) and IL-27β (1.93-fold) but to lesser extents (**Figure 6** **Bc**). L-lysine had no apparent effect on the expression of these genes (**Figure 6** **Bd**).

## Discussion

Water temperature alteration as a result of global warming can exacerbate bacterial diseases, such as edwardsiellosis, in fish, thus compromising farming productivity and sustainability (7, 24, 52–54). The causing agent of edwardsiellosis, *E. tarda*, senses the ambient temperature alteration *via* the PhoP-Q system and up-regulates the expression of virulence genes when the temperature reaches above 23°C (20, 26). In consistence, the occurrence of edwardsiellosis increases at high summer temperatures in farmed brook trout (*Salvelinus fontinalis*), channel catfish (*Ictalurus punctatus*) and rainbow trout (*Oncorhynchus mykiss*) (8). In the present work, we found that at 24 hpi, the dissemination of *E. tarda* in flounder was dramatically increased at 23°C compared with that at 15°C, indicating that, like the observations in other fish, higher temperature facilitated *E. tarda* infection in flounder. To examine whether metabolic change played a role in this process, comparative metabolomics was conducted at 24 hpi when the fish under both infection conditions (15°C and 23°C) were still alive and, therefore, metabolically and immunologically active.

Previous studies showed that bacterial infection induces profound metabolic reprograming in the host (32, 33). In this work, we analyzed the metabolomes of flounder in the absence and presence of *E. tarda* infection at 15°C and 23°C. Five SDMs were identified in the absence of bacterial infection (HC−LC), 59 SDMs were identified under the condition of *E. tarda* infection (HI−LI), and 57 SDMs were found to be present exclusively in HI−LI, which reflected the metabolic response of flounder to elevated temperature during *E. tarda* infection. These SDMs were highly enriched in amino acid metabolism pathways. Specifically, the bio-syntheses for L-valine, L-phenylalanine, L-arginine, L-aspartate, L-methionine, L-lysine, L-leucine, L-tryptophan, L-tyrosine, and L-threonine were significantly downregulated. In fish, amino acids constitute a major portion of energy substrates at relative high temperatures (55). For example, in Atlantic salmon (*Salmo solar*), amino acids, rather than glucose, were used for energy production upon chronical temperature elevation (56). In turbot (*Scophthalmus maximus*), the abundance of multiple amino acids significantly decreased in response to thermal stress (55). In our study, the extensive decrease of amino acids in flounder upon temperature elevation during infection implied a deficiency of amino acid-based energy source.

It has been recognized that, upon infection, host utilizes amino acid metabolism to restrict pathogen invasion and modulate the immune response (57, 58). For instance, L-arginine is the precursor of nitric oxide, an important bioactive molecule with both direct antimicrobial activity and immune signaling function (58). It can be converted to ornithine, the precursor of bioactive polyamines including putrescine, spermidine, and spermine, which have immune modulation activities (58). L-phenylalanine can be catabolized to phenylpyruvate, NH_3_, and H_2_O_2_, the latter product (H_2_O_2_) possesses direct bactericidal property, which is reinforced by the basification effect of NH_3_ (59). In view of the robust participation of amino acids in immune response, the decreased amino acid levels at high temperature observed in our study likely weakened the immune defense of flounder, resulting in elevated dissemination of *E. tarda*.

During *E. tarda* infection, the levels of succinate, aspartate, and arginosuccinate in flounder significantly decreased at high temperature. In mammals, succinate is an important mediator of immune signaling (60). Cytosolic accumulation of succinate leads to stabilization of the transcription factor hypoxia-inducible factor-1α (HIF-1α) and consequently induction of IL-1β expression, epigenetic alteration, and mitochondrial reactive oxygen species (ROS) production (30, 61). Moreover, succinate can be exported to the extracellular milieu, where it acts as the natural ligand for SUCNR1, a G protein–coupled receptor, and exerts a role akin to cytokine in inflammatory regulation (61). In M1 macrophages, which exhibits a metabolic shift in response to acute bacterial infection, the Krebs cycle was interrupted between succinate and fumarate, resulting in succinate accumulation (62). The broken Krebs cycle is re-fueled with fumarate by the aspartate-arginosuccinate shunt (63). In flounder, considering the enhanced infectivity of *E. tarda* at high temperature, succinate likely mediated a temperature-sensitive immunometabolic response against bacterial infection. In line with our observation, a recent study identified succinate as the crucial biomarker for phagocytosis in the monocytes/macrophages of Nile tilapia (*Oreochromis niloticus*), and exogenous succinate potentiated the phagocytosis of multiple bacteria including *E. tarda* (64).

Given the emerging role of metabolic intermediates in driving immune activation and regulation, it has been proposed that metabolism could be harnessed to combat severe pathogens such as multi-resistant bacteria and coronavirus (36, 65). In zebrafish (*Danio rerio*), tilapia (*Oreochromis niloticus*) and crucian carp (*Carassius auratus*), crucial metabolic biomarkers that enhanced host survival from bacterial infection were identified (5, 38, 66). In this study, we identified L-methionine and UDP-glucose as two effective metabolic biomarkers. L-methionine is known to supply methyl group through S-adenosylmethionine for the post-translational modification of immune effector proteins and nucleic acids, whereby facilitating cytokine expression in T cells and macrophages (67). In mice, chronic lung infection by *Pseudomonas aeruginosa* was cured by L-methionine in combination with antibiotics. In aquaculture fish species such as European seabass (*Dicentrarchus labrax*) and grass carp (*Ctenopharyngodon idella*), dietary methionine and methionine hydroxy analogue were found to be able to boost immunity and promote disease resistance (68, 69). UDP-glucose is a native agonist for P2Y_14_ receptor (P2Y_14_R), a G-protein–coupled receptor widely distributed in immune cells. Upon activation, P2Y_14_R inhibits adenylyl cyclase for the synthesis of cAMP, a second messenger participating in the signaling of various immune events such as inflammatory cytokine expression/secretion and pyroptosis (70, 71). In flounder, P2Y_14_R was reported to be involved in the inflammatory signaling (72). In the present work, we discovered that during *E. tarda* infection, L-methionine and UDP-glucose levels were significantly lower at 23°C than at 15°C, and exogenously added L-methionine and UDP-glucose effectively attenuated the infectivity of *E. tarda* and induced the expression of the proinflammatory cytokines of TNF-α, IL-1β, IL-6 and IL-27β. These findings suggest that L-methionine and UDP-glucose are able to improve the immune defense against *E. tarda* in flounder, thus highlighting the potential of L-methionine and UDP-glucose in the development of metabolic therapies against edwardsiellosis under the circumstances of global warming. Previous reports showed that diet deficiency of L-lysine enhanced the expression of proinflammatory cytokines in largemouth bass (*Micropterus salmoides*) and grass carp (*Ctenopharyngodon idella*) (73, 74). In this study, we found that exogenous L-lysine had no effect on the expression of TNF-α, IL-1β, IL-6, IL-8 or IL-27β in flounder leukocytes. The ineffectiveness of L-lysine could be due to a number of factors, such as the dose, the species and the physiological status of the fish, and the time of examination.

In conclusion, the present study profiled the temperature effect on the metabolic response of flounder to *E. tarda* infection and identified the biomarkers that promoted host resistance to *E. tarda* at high temperature. Our results revealed the existence of temperature-regulated metabolic reprogramming in fish during bacterial infection, and highlighted an increasing risk of fish disease caused by water temperature elevation as a foreseeable consequence of global warming. In addition, our study also suggested a potential for the development of metabolic reengineering therapy against fish disease.

## Data availability statement

The original contributions presented in the study are included in the article/**Supplementary Material**. Further inquiries can be directed to the corresponding authors.

## Ethics statement

The animal study was reviewed and approved by Ethics Committee of Institute of Oceanology, Chinese Academy of Sciences.

## Author contributions

BiS: investigation, writing - original draft. BoS: conceptualization, investigation, writing - original draft, writing - review and editing. BZ: investigation. LS: conceptualization, supervision, funding acquisition, writing - review and editing. All authors contributed to the article and approved the submitted version.

## Funding

This work was financed by the grants of the National Key R&D Program of China (2018YFD0900500), the National Natural Science Foundation of China (41876170), and the Taishan Scholar Program of Shandong Province.

## Conflict of interest

The authors declare that the research was conducted in the absence of any commercial or financial relationships that could be construed as a potential conflict of interest.

## Publisher’s note

All claims expressed in this article are solely those of the authors and do not necessarily represent those of their affiliated organizations, or those of the publisher, the editors and the reviewers. Any product that may be evaluated in this article, or claim that may be made by its manufacturer, is not guaranteed or endorsed by the publisher.
