# Supplementary material for: Temperature induces metabolic reprogramming in fish during bacterial infection
Source: Front Immunol. 2022 Sep 15;13:1010948. doi: 10.3389/fimmu.2022.1010948 (PMC9520329; doi:10.3389/fimmu.2022.1010948)
Supplement: Supplementary file 1 [file DataSheet_1.docx]

**Supplementary Materials**

**FIGURE S1** The effect of temperature on *Edwardsiella tarda* infection. **(A)** The time-course acclimation of flounder to 15°C and 23°C. **(B)** The acclimatized fish were infected with *E. tarda*, and bacterial dissemination in spleen was determined at 24 h post-infection. **, *p* < 0.01.


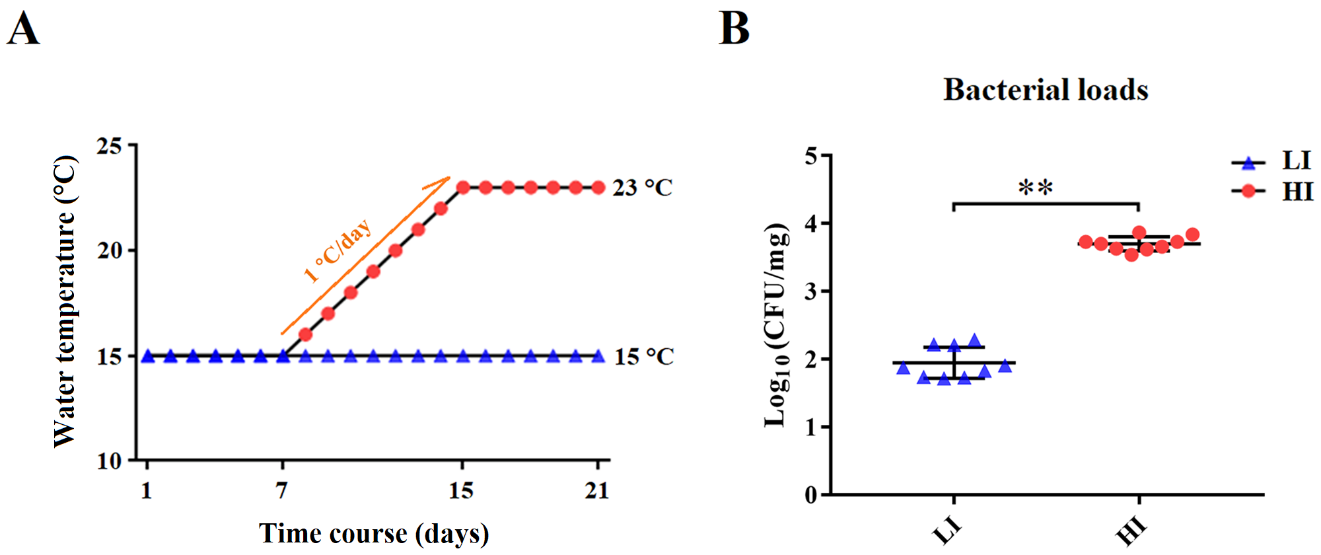


**FIGURE S2** Z scores (standard deviation from average) of metabolites in LI−LC (A) and HI−HC (B) correspond to the data shown in FIGURE 2A and FIGURE 2B, respectively. Each point represents one biological repeat for the metabolite. ★, metabolites occurring in both LI−LC and HI−HC.


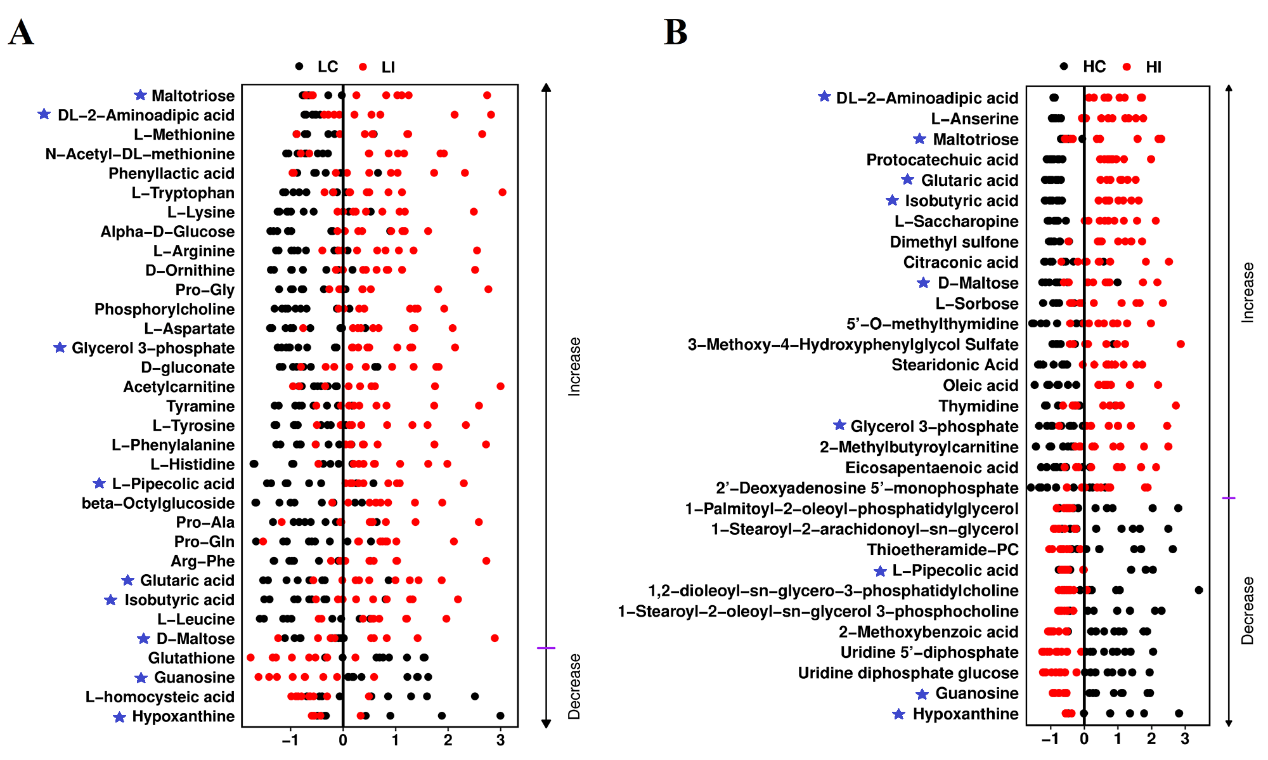


**FIGURE S3** Top 10 KEGG enrichment of the SDMs of LI−LC **(A)** and HI−HC **(B)**.


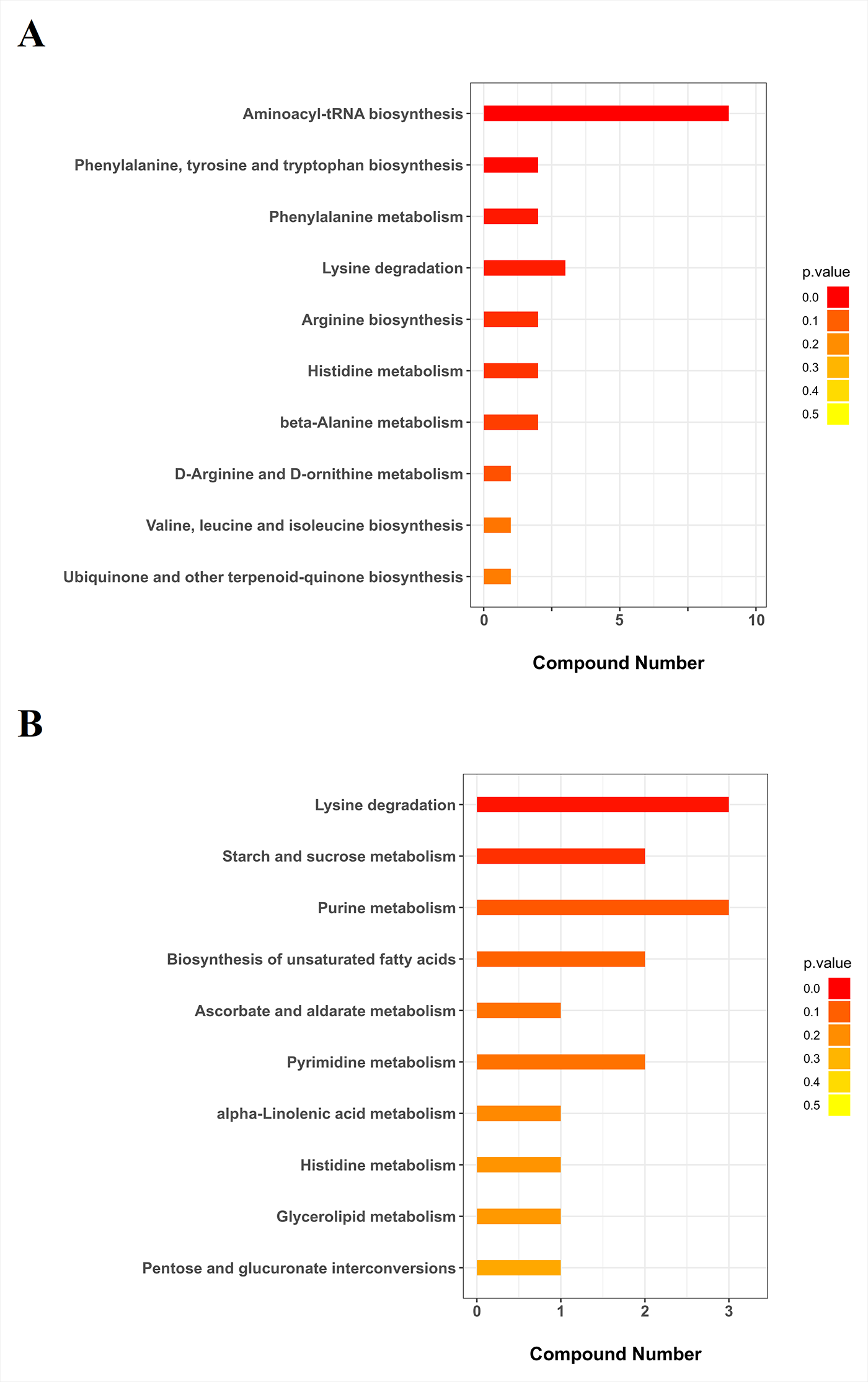


**FIGURE S4** Venn diagram of the common and unique SDMs in HC−LC (blue circle) and HI−LI (pink circle).


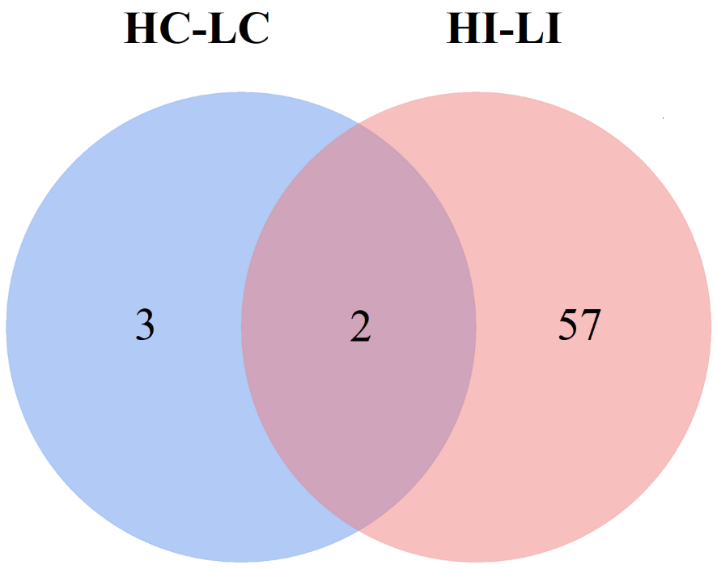


**FIGURE S5** Z scores (standard deviation from average) of metabolites in HC−LC (A) and HI−LI (B) correspond to the data shown in FIGURE 3A and FIGURE 3B, respectively. Each point represents one biological repeat for the metabolite. ★, metabolites occurring in both HC−LC and HI−LI.


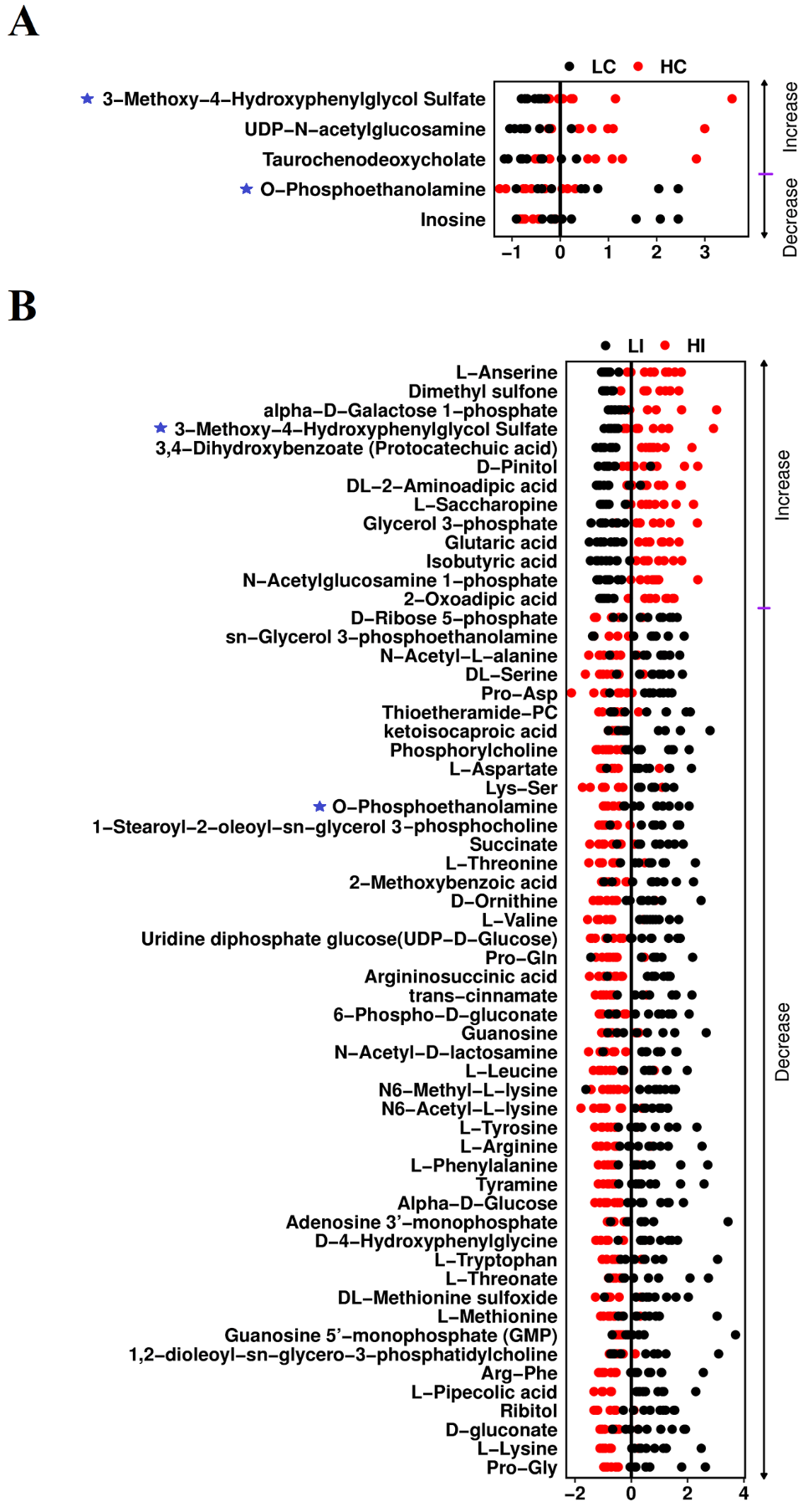


**FIGURE S6** Categories of the unique SDMs in HI−LI. The category percentage **(A)** and number variation **(B)** of the SDMs in HI−LI are shown.


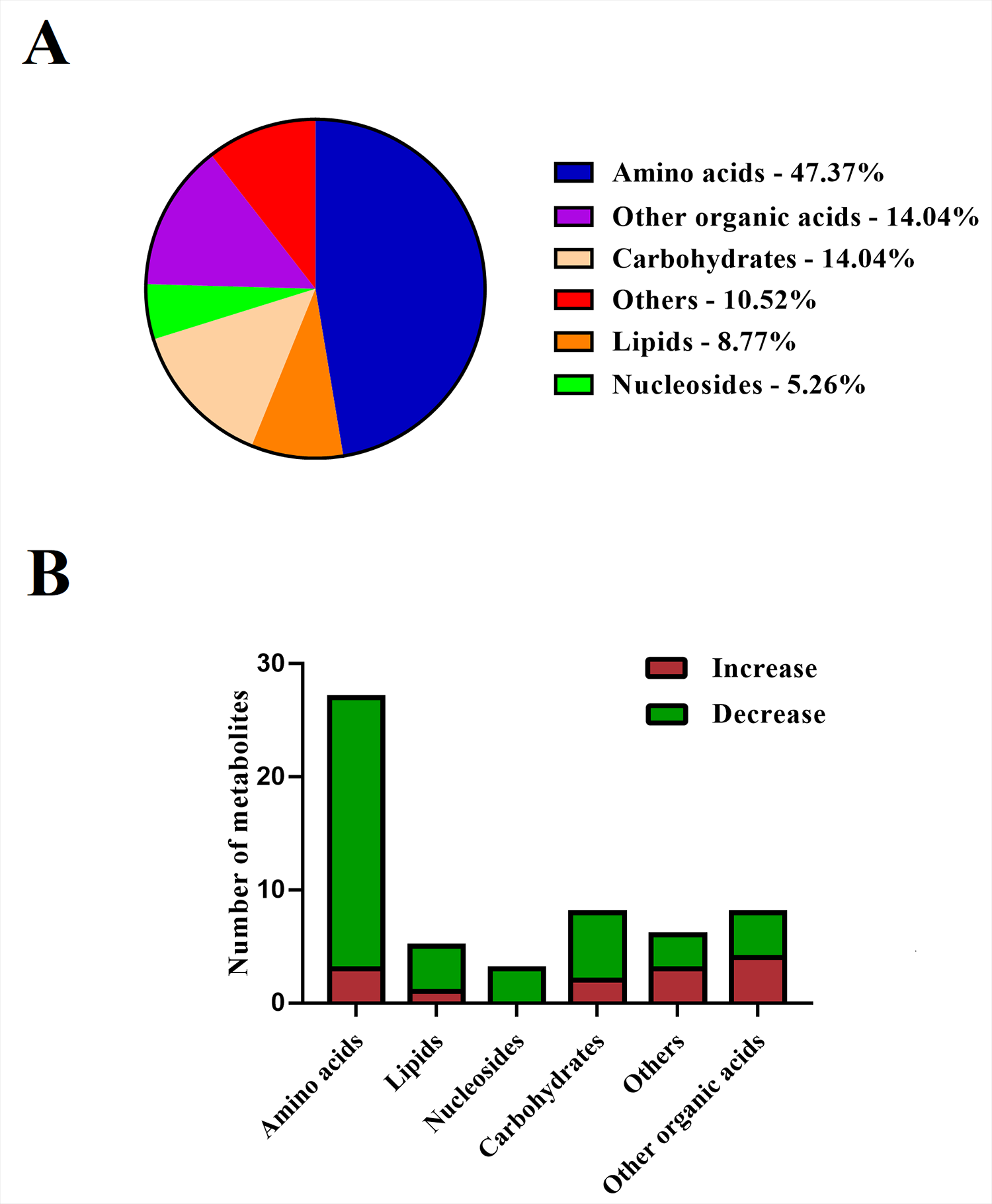


**FIGURE S7** KEGG enrichment (top 10) of the 22 SDMs shared between LI−LC and HI−LI.


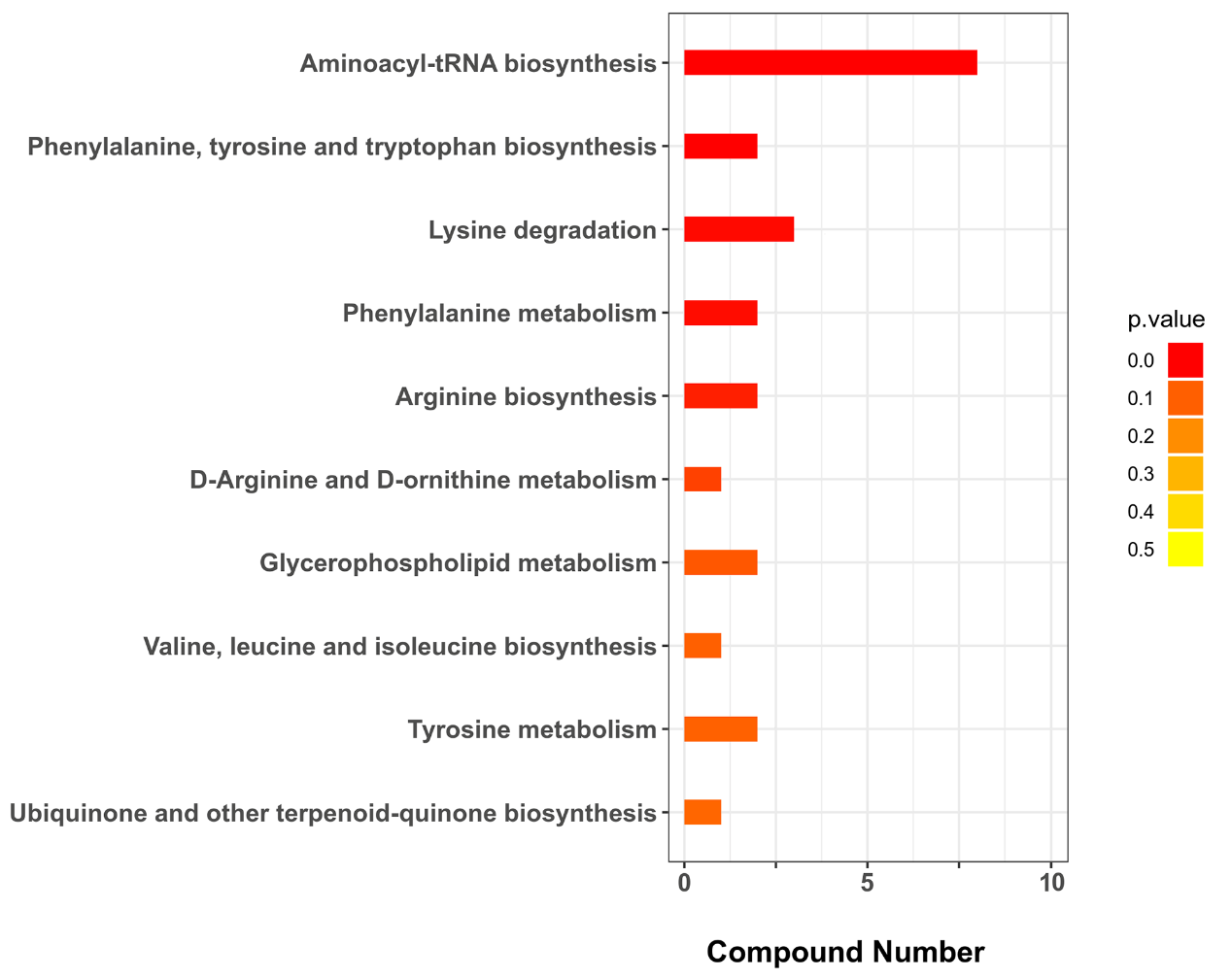


**FIGURE S8** KEGG enrichment (top 10) of 16 SDMs shared between HI−HC and HI−LI.


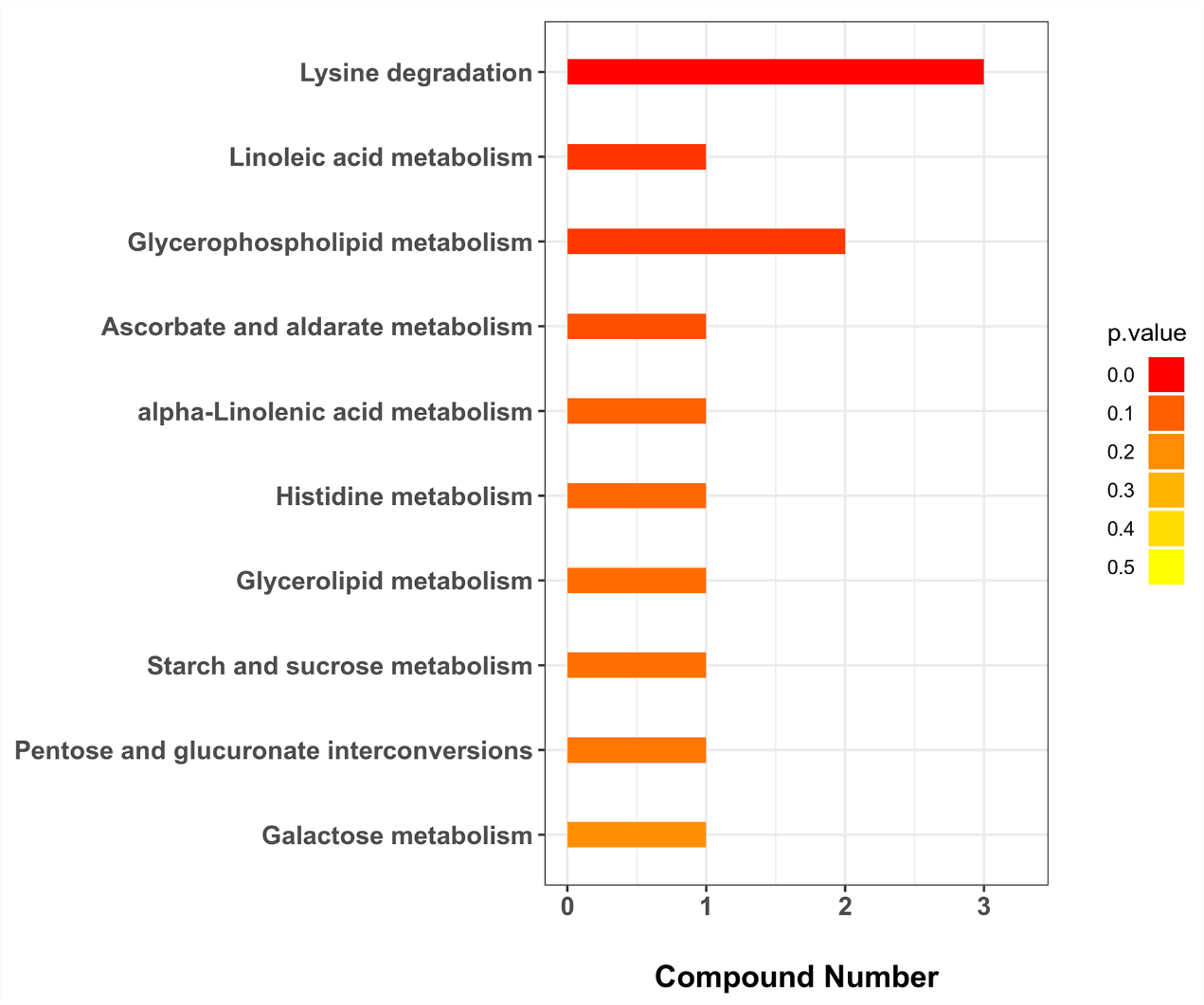


**TABLE S5** Top 10 KEGG enriched pathways of the SDMs in LI−LC. The *p*-value was set as < 0.05. Up and down arrows indicate upregulation and downregulation, respectively.

| **Metabolic pathway** | **Hits** | ***p*-value** | **Metabolites** |
| --- | --- | --- | --- |
| Aminoacyl-tRNA biosynthesis | 9 | 1.03E-07 | L-Histidine (↑), L-Phenylalanine (↑),  L-Arginine (↑), L-Aspartate (↑),  L-Methionine (↑), L-Lysine (↑),  L-Leucine (↑), L-Tryptophan (↑),  L-Tyrosine (↑) |
| Phenylalanine, tyrosine and tryptophan biosynthesis | 2 | 2.15E-03 | L-Phenylalanine (↑), L-Tyrosine (↑) |
| Phenylalanine metabolism | 2 | 9.54E-03 | L-Phenylalanine (↑), L-Tyrosine (↑) |
| Lysine degradation | 3 | 1.14E-02 | L-Lysine (↑), DL-2-Aminoadipic acid (↑), L-Pipecolic acid (↑) |
| Arginine biosynthesis | 2 | 2.88E-02 | L-Arginine (↑), L-Aspartate (↑) |
| Histidine metabolism | 2 | 3.29E-02 | L-Histidine (↑), L-Aspartate (↑) |
| beta-Alanine metabolism | 2 | 4.62E-02 | L-Histidine (↑), L-Aspartate (↑) |
| D-Arginine and D-ornithine metabolism | 1 | 7.58E-02 | D-Ornithine (↑), |
| Valine, leucine and isoleucine biosynthesis | 1 | 1.46E-01 | L-Leucine (↑) |
| Ubiquinone and other terpenoid-quinone biosynthesis | 1 | 1.63E-01 | L-Tyrosine (↑) |

**TABLE S6** Top 10 KEGG enriched pathways of the SDMs in HI−HC. The *p*-value was set as < 0.05. Up and down arrows indicate upregulation and downregulation, respectively.

| **Metabolic pathway** | **Hits** | ***p*-value** | **Metabolites** |
| --- | --- | --- | --- |
| Lysine degradation | 3 | 6.55E-03 | L-Saccharopine (↑), DL-2-Aminoadipic acid (↑), L-Pipecolic acid (↓) |
| Starch and sucrose metabolism | 2 | 2.88E-02 | UDP-Glucose (↓), D-Maltose (↑) |
| Purine metabolism | 3 | 8.50E-02 | 2'-Deoxyadenosine 5'-monophosphate (↑), Hypoxanthine (↓), Guanosine (↓) |
| Biosynthesis of unsaturated fatty acids | 2 | 1.06E-01 | Oleic acid (↑), Eicosapentaenoic acid (↑) |
| Ascorbate and aldarate metabolism | 1 | 1.36E-01 | UDP-Glucose (↓) |
| Pyrimidine metabolism | 2 | 1.38E-01 | UDP (↓), Thymidine (↑) |
| alpha-Linolenic acid metabolism | 1 | 1.90E-01 | Stearidonic acid (↑) |
| Histidine metabolism | 1 | 2.16E-01 | L-Anserine (↑) |
| Glycerolipid metabolism | 1 | 2.29E-01 | Glycerol 3-phosphate (↑) |
| Pentose and glucuronate interconversions | 1 | 2.65E-01 | UDP-Glucose (↓) |

**TABLE S7** Top 10 KEGG enriched pathways of the 22 SDMs shared between LI−LC and HI−LI. The *p*-value was set as < 0.05. Up and down arrows indicate upregulation and downregulation, respectively.

| **Metabolic pathway** | **Hits** | ***p*-value** | **Metabolites (LI**−**LC, HI**−**LI)** |
| --- | --- | --- | --- |
| Aminoacyl-tRNA biosynthesis | 8 | 4.89E-08 | L-Phenylalanine (↑, ↓), L-Arginine (↑, ↓), L-Aspartate (↑, ↓), L-Methionine (↑, ↓), L-Lysine (↑, ↓), L-Leucine (↑, ↓),  L-Tryptophan (↑, ↓), L-Tyrosine (↑, ↓) |
| Phenylalanine, tyrosine and tryptophan biosynthesis | 2 | 9.79E-04 | L-Phenylalanine (↑, ↓), L-Tyrosine (↑, ↓) |
| Lysine degradation | 3 | 3.75E-03 | L-Lysine (↑, ↓), DL-2-Aminoadipic acid (↑, ↑), L-Pipecolic acid (↑, ↓) |
| Phenylalanine metabolism | 2 | 4.43E-03 | L-Phenylalanine (↑, ↓), L-Tyrosine (↑, ↓) |
| Arginine biosynthesis | 2 | 1.37E-02 | L-Arginine (↑, ↓), L-Aspartate (↑, ↓) |
| D-Arginine and D-ornithine metabolism | 1 | 5.19E-02 | D-Ornithine (↑, ↓) |
| Glycerophospholipid metabolism | 2 | 8.78E-02 | Phosphorylcholine (↑, ↓), Glycerol 3-phosphate (↑, ↑) |
| Valine, leucine and isoleucine biosynthesis | 1 | 1.01E-01 | L-Leucine (↑, ↓) |
| Tyrosine metabolism | 2 | 1.04E-01 | L-Tyrosine (↑, ↓), Tyramine (↑, ↓) |
| Ubiquinone and other terpenoid-quinone biosynthesis | 1 | 1.13E-01 | L-Tyrosine (↑, ↓) |

**TABLE S8** Top 10 KEGG enriched pathway of the 16 SDMs shared between HI−HC and HI−LI. The *p*-value was set as < 0.05. Up and down arrows indicate upregulation and downregulation, respectively.

| **Metabolic pathway** | **Hits** | ***p*-value** | **Metabolites (HI**−**HC, H**−**LI)** |
| --- | --- | --- | --- |
| Lysine degradation | 3 | 9.24E-04 | L-Saccharopine (↑, ↑), DL-2-Aminoadipic acid (↑, ↑), L-Pipecolic acid (↓, ↓) |
| Linoleic acid metabolism | 1 | 3.30E-02 | 1,2-dioleoyl-sn-glycero-3-phosphatidylcholine (↓, ↓) |
| Glycerophospholipid metabolism | 2 | 3.80E-02 | 1,2-dioleoyl-sn-glycero-3-phosphatidylcholine (↓, ↓), Glycerol 3-phosphate (↑, ↑) |
| Ascorbate and aldarate metabolism | 1 | 7.29E-02 | UDP-glucose (↓, ↓) |
| alpha-Linolenic acid metabolism | 1 | 1.04E-01 | 1,2-dioleoyl-sn-glycero-3-phosphatidylcholine (↓, ↓) |
| Histidine metabolism | 1 | 1.19E-01 | L-Anserine (↑, ↑) |
| Glycerolipid metabolism | 1 | 1.26E-01 | Glycerol 3-phosphate (↑, ↑) |
| Starch and sucrose metabolism | 1 | 1.34E-01 | UDP-glucose (↓, ↓) |
| Pentose and glucuronate interconversions | 1 | 1.48E-01 | UDP-glucose (↓, ↓) |
| Galactose metabolism | 1 | 2.04E-01 | UDP-glucose (↓, ↓) |
